# Breaking Barriers: Transitioning from X-ray Crystallography to Cryo-EM for Structural Studies of ATAD2B

**DOI:** 10.1101/2025.10.13.682238

**Published:** 2025-10-14

**Authors:** Hassan Zafar, Kiera L. Malone, Ajit K. Singh, Michael A. Cianfrocco, Karen C. Glass

**Affiliations:** 1 Department of Pharmacology, Larner College of Medicine, University of Vermont, Burlington, VT, 05405, USA; 2 Department of Biochemistry, Larner College of Medicine, University of Vermont, Burlington, VT, 05405, USA; 3 University of Vermont Cancer Center, University of Vermont, Burlington, VT, 05405, USA; 4 Life Sciences Institute, Department of Biological Chemistry, University of Michigan, Ann Arbor, Michigan, 48109, USA

**Keywords:** Cryo-electron microscopy (cryo-EM), Structural biology, Single-particle analysis, ATAD2B, Bromodomain, GroEL, Macromolecular X-ray crystallography, Protein expression and purification, Data processing workflows, Model building and refinement

## Abstract

Cryo-electron microscopy (cryo-EM) has transformed structural biology by enabling near-atomic resolution of large macromolecular complexes without the need for crystallization. Here, we describe our laboratory’s transition from X-ray crystallography to single-particle cryo-EM to investigate the ATPase family AAA+ domain-containing protein 2B (ATAD2B), a chromatin regulator implicated in epigenetic signaling. We outline the challenges encountered during protein expression, purification, and sample preparation, including co-purification of the chaperonin GroEL, and strategies employed to overcome these obstacles. Our workflow highlights critical steps in sample optimization, grid vitrification, and data processing using CryoSPARC, cisTEM, and Topaz, as well as computational requirements for high-resolution reconstructions. We also discuss model building, refinement, and validation approaches, emphasizing best practices for new cryo-EM users. This work provides practical insights for structural biologists adopting cryo-EM, particularly for large, flexible protein complexes, and underscores the importance of integrated approaches combining biochemical, computational, and imaging strategies.

## 1. The Expanding Toolkit of Structural Biology

The landscape of structural biology is constantly evolving, and significant advancements in cryo-electron microscopy (cryo-EM) methodologies have enabled us to study large macromolecular complexes of proteins and nucleic acids at near atomic resolution (Chua et al., 2022). Structural biology techniques such as X-ray crystallography, nuclear magnetic resonance, and electron microscopy have provided invaluable insights into the molecular machines that drive biological processes in the cell (Shoemaker & Ando, 2018). X-ray crystallography has long been the backbone of high-resolution structure determination, offering exquisite detail when well-diffracting crystals are available (Smyth & Martin, 2000, Zheng et al., 2015). However, the crystallization of many target biomolecules, particularly membrane proteins and large multicomponent complexes, has remained a challenge (Birch et al., 2018).

**Figure 1.**
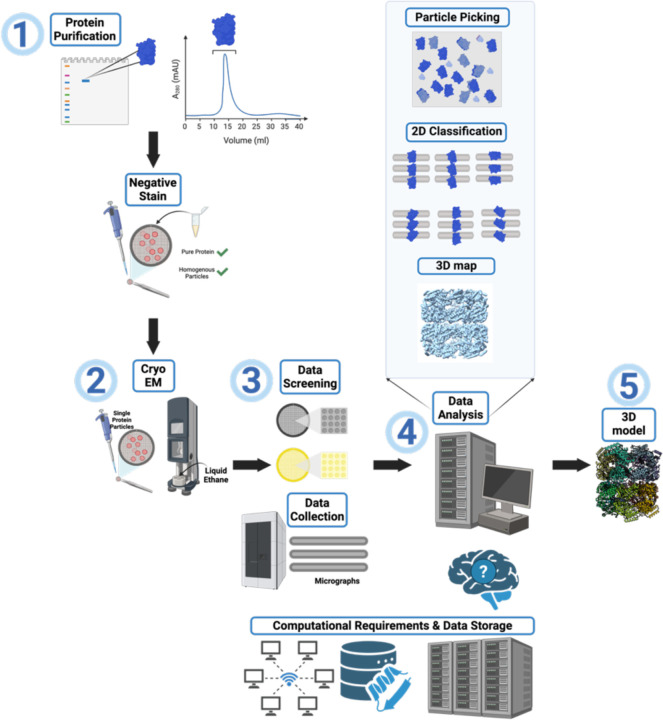
Overview of the cryo-EM workflow. Step 1: Evaluation of protein sample purity and quality to determine suitability for structural characterization by cryo-EM. Step 2: Vitrification of samples for cryo-EM imaging by applying the purified protein to a cryo-EM grid followed by plunge freezing. Step 3: Screening of vitrified cryo-EM grids to identify optimal freezing conditions for the purified sample. Parameters such as grid type, buffer components, additives, and plunge-freezing time/force are assessed. Once sample preparation is optimized, larger cryo-EM datasets are collected. Step 4: Data processing using specialized software, including motion correction, CTF estimation, particle picking, 2D classification, and 3D refinement, to obtain a high-resolution 3D map. Step 5: Model building and refinement to fit a model into the map. Model validation is performed to assess the quality of the map and the model fit. *Note: The cryo-EM workflow often requires iterative optimization at multiple stages, and users may need to revisit earlier steps to achieve a final structure at the desired resolution.*

In cryo-EM, biological samples are applied onto specialized grids, which are then rapidly plunge-frozen (Thompson et al., 2016). Techniques like single-particle analysis have allowed researchers to achieve resolutions comparable to X-ray crystallography, while also revealing structural heterogeneities and dynamic intermediate states of molecular pathways (Cheng, 2015). However, the initial bottlenecks of employing cryo-EM include large investments in highly specialized electron microscopes and computational infrastructure (Meng et al., 2023). Additionally, the cryo-EM workflow, as illustrated in Figure 1, can be quite daunting.

Often, researchers must use an integrated approach, combining multiple techniques to obtain structural information on large biomolecular complexes (Nogales & Scheres, 2015). For instance, studies on large ribonucleoprotein complexes used the high-resolution information garnered with X-ray crystallography for individual subunits in conjunction with lower resolution cryo-EM maps of the multimeric complex to generate composite models (Bai et al., 2015). This synergy not only validates the individual findings of each method but also creates a more robust picture of the larger molecular architecture, which provides information that is essential for our understanding of biological mechanisms, and for the development of novel therapeutic interventions (Bijak et al., 2023).

## 2. Rationale for Developing Expertise in Cryo-EM for Structure Determination

### Why Cryo-EM is Critical for Studying ATAD2B

In the Glass laboratory, we are interested in understanding how epigenetic signaling regulates gene expression and how alterations in these pathways are involved in disease development. Our research on bromodomain-containing proteins has focused on revealing the molecular mechanisms driving recognition of post-translational modifications found on histone proteins in the nucleosome. Bromodomains are a conserved structural motif that function as a chromatin reader domain to recognize acetylated lysine residues on histone proteins (Zeng & Zhou, 2002). Binding to specific acetyllysine modifications on histones often bridges the associated bromodomain-containing protein to chromatin where it can carry out its molecular activities. For the past several years our research has focused on the human ATPase family AAA+ domain-containing protein 2 (ATAD2), and its closely related paralog ATAD2B (Gay et al., 2019, Lloyd et al., 2020, Evans et al., 2021, Phillips, Malone, et al., 2024). We have employed a variety of approaches including structural biology, molecular biology, genomics, biochemistry, biophysics, and proteomics to determine how physiologically abundant combinations of histone modifications regulate the reader activity of the ATAD2 and ATAD2B bromodomains. However, how the bromodomain region contributes to the cellular and molecular activities of ATAD2 and ATAD2B is unknown. These proteins are thought to function as regulators of chromatin architecture (Morozumi et al., 2016, Koo et al., 2016, Lazarchuk et al., 2020), and we hypothesized that recognition of acetyllysine residues in the flexible N-terminal region of histones plays an important role in directing the AAA+ ATPase activity of ATAD2B to its chromatin substrates. We quickly realized that using a structure-function approach to understand how ATAD2B contributes to these fundamental cellular mechanisms, designed to maintain chromatin organization, would necessitate isolating the full-length protein to study its activity *in vitro*.

**Figure 2.**
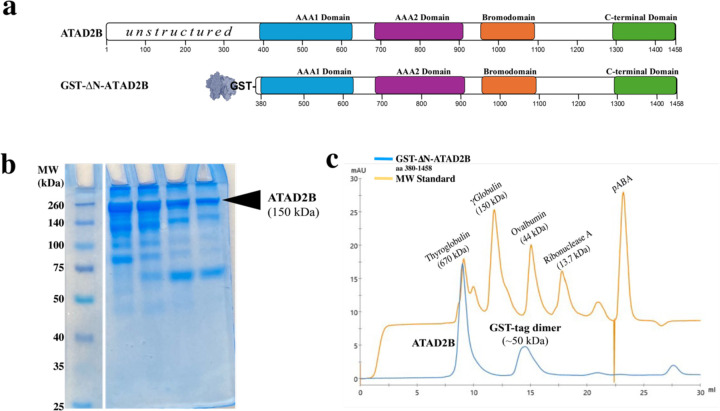
Evaluation of ATAD2B oligomerization. a) Domain organization of the full-length human ATAD2B protein (aa 1–1458), and the GST-tagged, N-terminally truncated-ATAD2B expression construct (aa 380–1458). This GST-∆N-ATAD2B (aa 380–1458) construct is referred to as ATAD2B for the remainder of the text for clarity. b) 10% SDS-PAGE gel stained with Coomassie blue showing fractions collected during ATAD2B purification. ATAD2B is the top band that runs above its expected molecular weight of 150 kDa. Additional proteins in the same lane indicate the presence of heterogeneity in the ATAD2B protein sample. c) Size exclusion chromatography traces using a Superdex 200 Increase 10/300 GL column on an ÄktaPrime system (GE/Cytiva) for ATAD2B (blue) and a molecular weight standard mixture (orange). The expected molecular weight of monomeric ATAD2B is 150 kDa. The elution profile shows the first peak is consistent with hexameric ATAD2B, which elutes prior to thyroglobulin (670 kDa), the second peak is consistent with a dimerized GST tag (50 kDa), eluting prior to ovalbumin at (44 kDa).

The ATAD2B protein is 1,458 amino acid residues long and contains an unstructured N-terminal region, two AAA+ ATPase domains, a bromodomain, and an ordered C-terminal domain (Figure 2a) (Puchades et al., 2020, Khan et al., 2022). Previous studies on human ATAD2, and a yeast homolog Abo1, indicated that truncating the unstructured N-terminus would increase our ability to successfully express and purify the ATAD2B protein for biochemical and structural studies, without significantly impacting its enzymatic ATPase function (Cho et al., 2023, Cho et al., 2019). Thus, we codon optimized the DNA encoding the human ATAD2B protein residues 380–1458 for expression in *E. coli* and inserted it into the pGEX-6P-1 vector to add an N-terminal GST-affinity tag. We started with our well-established system in *E. coli* due to its ease of use and our previous experience purifying the ATAD2B bromodomain. However, the 150 kDa GST-∆N-ATAD2B, res 380–1458 construct was considerably larger than any other protein we purified previously (Singh et al., 2022, Poplawski et al., 2014, Phillips, Malone, et al., 2024, Obi et al., 2020, Lubula, Eckenroth, et al., 2014, Lubula, Poplawaski, et al., 2014, Lloyd et al., 2020, Gay et al., 2019, Evans et al., 2021). For the remainder of the text, this GST-∆N-ATAD2B construct (residues 380–1458) will be referred to as ATAD2B for clarity. Not surprisingly, the expression of ATAD2B in *E. coli* was very low, but after optimizing several factors including the IPTG concentration, buffer components, and the *E. coli* cell line we were able to reliably obtain ~500 μL of 2.5 mg/mL ATAD2B from a four liter culture. However, there was a considerable amount of contaminating proteins remaining in the sample, even after the GST affinity and size exclusion chromatography columns (Figure 2b).

This sample was not pure enough, or in the milligram quantities needed for the X-ray crystallization approach we used previously with the ATAD2 and ATAD2B bromodomains (Phillips, Malone, et al., 2024, Lubula, Eckenroth, et al., 2014, Lubula, Poplawaski, et al., 2014, Lloyd et al., 2020, Gay et al., 2019, Evans et al., 2021). Additionally, the 150 kDa ATAD2B AAA+ ATPase was expected to hexamerize into a ~900 kDa complex upon ATP nucleotide binding (Puchades et al., 2020, Khan et al., 2022), and this size is well above the upper limit for structure determination using solution NMR, which is another structural determination approach we are familiar with (Poplawski et al., 2014, Kim et al., 2016, Obi et al., 2020, Evans et al., 2021, Singh et al., 2022, Phillips, Cook, et al., 2024). The structure of the closely related yeast homolog of ATAD2B (Abo1) was determined using cryo-EM (Cho et al., 2019), which prompted us to begin learning more about this technique by reaching out to the National cryo-EM Access and Training Center (NCCAT) in New York, NY. We first heard about the NCCAT from attending structural biology meetings such as the American Crystallographic Association (ACA) and the Biophysical Society, along with word of mouth from colleagues. The NCCAT staff pointed us to several online cryo-EM resources (Table 1), including the Caltech Getting Started in cryo-EM course that includes lecture videos by Dr. Grant Jensen (Technology, n.d., Course, 2013). Fortuitously, we also attended the ACA annual meeting in Portland, OR, where we participated in a workshop on ‘Hands-on Single-Particle CryoEM Data Analysis with cryoEDU’ that was organized by Dr. Michael Cianfrocco. Additionally, a large portion of the scientific sessions at that meeting were focused on cryo-EM, including “New developments in cryo-EM”, “Machine learning in cryo-EM”, and “Structures of very large assemblies”.

The ACA meeting exposed us to the capabilities of cryo-EM, and we quickly discovered that cryo-EM requires significantly less protein than crystallography, that it can tolerate heterogenous samples, and that the ATAD2B AAA+ ATPase complex is an ideal size for structure determination using this method (Venien-Bryan et al., 2017). While structural biology techniques such as X-ray Crystallography and NMR require milligram quantities of highly purified protein samples to obtain high-quality/high-resolution data, it appeared to us that cryo-EM could handle sample heterogeneity a bit better (Takizawa et al., 2017, Wang & Wang, 2017), thanks to the downstream data processing workflow. Therefore, we decided it would be worthwhile to pursue learning cryo-EM to study the structure and function of human ATAD2B.

Early in this process, a postdoctoral trainee in the Glass laboratory had the opportunity to attend the cryo-EM short course at the NCCAT. As part of this course they were able to image the ATAD2B protein sample using negative stain microscopy (Figure 1, step 1), and the NCCAT staff also thought ATAD2B would be a good candidate to study using cryo-EM methods. To move forward we needed access to a plunge freezing system and a TEM microscope to make and screen cryo-EM grids. However, this expensive and specialized equipment was not available locally, so we applied to the TP1 training program at the NCCAT in NYC. To gain comprehensive, hands-on experience with every stage of cryo-EM sample preparation and data processing, four members of the Glass Lab traveled in pairs to the NCCAT for weeklong visits each month over the course of a year. When each team of two returned, they shared what was learned and their experiences troubleshooting with the rest of the lab. This allowed us to maximize our time with the NCCAT experts to learn the techniques necessary to determine the structure of the ATAD2B complex.

### Helpful Resources for when you decide cryo-EM is right for you

For those interested in or new to cryo-EM, we highly recommend viewing introductory videos on cryo-EM theory and sample preparation. There are a plethora of different resources, and we have compiled many of them in Table 1. Additionally, virtual seminars, webinars, and scientific meetings are a great place to begin learning about cryo-EM. For those who want to learn more about cryo-EM facilities located nearby prior to investing in this technique, we would encourage readers to read the information available at each National Cryo-EM Center that are funded by the NIH-NIGMS. Moreover, Pacific Northwest cryo-EM Center (PNCC) has created a global list of available cryo-EM centers (see Table 1). We also encourage readers to visit the interactive map of ‘HighEnd CryoEM Worldwide’ that displays type of microscope, camera, and operation style, if appliable, in addition to contact information (Table 1). Reaching out to a facility directly will allow you to learn if the services they offer will fit your needs. For example, the national cryo-EM centers provide on-site training in addition to core services, whereas more regional institutions may accept submission of a purified protein sample, or a protein sample frozen on a grid.

## 3. Acquiring Technical Skills for Sample Preparation and Optimization

The transition from X-ray crystallography to cryo-EM involves modifications in sample preparation protocols, as the requirements for optimal imaging differ between the two techniques (Stark & Chari). In X-ray crystallography, the main goal is coaxing proteins or nucleic acids to form well-ordered crystals (Smyth & Martin, 2000). This requires optimization of various parameters such as pH, temperature, precipitating agents, and chemical additives—to discover the optimal conditions that promote crystal lattice formation. Well-ordered crystals with good diffraction yield high-resolution structural data (McPherson & Cudney, 2014).

For single-particle cryo-EM experiments, crystallization of samples is not required, though proteins and nucleic acids are often purified using conventional approaches (Saibil, 2022). Purified samples are applied to cryo-EM grids, which are typically coated with an ultra-thin film made from materials such as carbon, gold, or graphene. These films serve as a supportive surface for the biomolecules, helping to anchor them in place and maintain their native conformation. The film also facilitates the formation of a thin layer of vitreous ice during plunge freezing, which is essential for capturing biomolecules in a near-native state and preserving the sample’s structural integrity under the electron beam. The choice of film material can influence particle distribution, orientation, and contrast, and is often tailored to the specific requirements of the experiment (Lyumkis, 2019). Extensive optimization of the sample freezing conditions is often necessary to achieve uniform particle distribution and the desired ice thickness on the grid, both of which are critical for obtaining high-quality cryo-EM micrographs (Table 2) (Han et al., 2023). The quality of the cryogrid sample directly impacts the success of downstream imaging and data analysis, thus making this step as critical in cryo-EM as crystallization is in X-ray studies.

Transitioning to cryo-EM demands not only technical skill in operating transmission electron microscopes, but also familiarity with the specialized software used for data collection and analysis. Researchers must become adept at adjusting a wide range of settings—such as electron beam parameters, detector configurations, and imaging conditions—all of which play a crucial role in determining the quality of the resulting data. This involves understanding complex concepts such as defocus optimization, astigmatism correction, and dose management to minimize beam damage while maximizing signal quality. Additionally, practitioners must assess ice quality and particle distribution to optimize data collection strategies. This technical expertise represents a substantial investment in skill development that is essential for successful cryo-EM research.

### Gaining expertise in electron microscopy

Transitioning from X-ray crystallography to cryo-EM is like stepping into a new world, but one that speaks a familiar language. Our structural studies on ATAD2B began with crystallization attempts, which failed due to the inability to obtain high concentrations of purified ATAD2B. This challenge led us to pursue cryo-EM as an alternative approach. We initiated our efforts with negative staining to evaluate sample homogeneity, integrity, and concentration, key indicators for cryo-EM readiness using the JEOL 1400 TEM, 120 keV microscope, equipped with an AMT XR611 CCD camera available through the Microscopy Imaging Center at the University of Vermont Larner College of Medicine. As we lacked in-house vitrification tools, we brought the ATAD2B sample to NCCAT for plunge-freezing. There, we quickly realized that the sample conditions for cryogrid preparation is as critical as buffer optimization for crystal growth. Variables such as salt, detergent, and glycerol can dramatically affect vitrification quality and particle distribution. High protein concentrations can lead to aggregation, while low concentrations result in sparse particle visibility. Grid freezing introduces another level of complexity. Parameters like blotting time, blot force, temperature, and humidity all required optimization. Even the type of grid significantly influenced particle orientation and distribution (Weissenberger et al., 2021, Wang & Zimanyi, 2024). After testing multiple conditions, we found that a buffer containing 50 mM Tris (pH 7.5), 150 mM NaCl, and 5% glycerol, vitrified on a 1.2/1.3 UltrAuFoil grid using a Vitrobot Mark IV, provided the best results in terms of particle orientation and distribution. Every step from ice thickness to grid clipping required precise, careful handling. These seemingly minor details were essential to enable successful high-resolution cryo-EM imaging.

### Computational Requirements are an important factor for cryo-EM datasets

cryo-EM data processing involves the handling of large datasets and requires substantial computational resources to perform key tasks such as motion correction, CTF estimation, particle picking, 2D classification, and 3D refinement (Baldwin et al., 2018). For any lab entering the cryo-EM field, one of the first and most critical questions is which data processing suite(s) should you use and how do we meet the computational demands of these programs? When we began transitioning into cryo-EM, we faced the same challenge-should we invest in our own GPU workstations, rely on a cloud-based service, use a national shared facility, or work with the university’s high-performance computing (HPC) cluster? After evaluating our needs and budget constraints, we opted to use the Vermont Advanced Computing Core (VACC) , which offered a cost-effective and scalable solution with routine data backups. To assist others in navigating this decision, we have compiled a practical summary of the core computational requirements and commonly used software suites and platforms in Table 3.

## 4. Common Pitfalls and Problems

### ATAD2B sample heterogeneity caused by a common contaminating protein

Early on in our data processing workflow, we observed heptameric particles in both our micrographs and 2D class averages. However, the cryo-EM structures of ATAD2 homologs were all hexameric (Cho et al., 2019, Cho et al., 2023, Wang et al., 2023). Initially, we hypothesized that these heptameric particles may support a new function for ATAD2B (Cho et al., 2019, Cho et al., 2023, Wang et al., 2023). To ensure that the heptameric particles were indeed ATAD2B, and not some unwanted protein, we sent both a liquid sample and cutout bands from the SDS-PAGE gel of the ATAD2B sample shown in Figure 2b to a mass spectrometry core. In each sample, human ATAD2B was present. Because we were pushing the size limit for soluble protein expression in *E. coli* (Chae et al., 2017), these results confirmed previously held suspicions that this heterogeneity may be coming from sample degradation or internal cleavage of the ATAD2B protein. The aforementioned positive results gave us the confidence to continue with high resolution data collection. Our goal was to collect data on ATAD2B bound in three different nucleotide states to capture any large secondary/tertiary structural confirmational changes that occur between these states, and gain information on how nucleotide coordination changes within the binding pocket.

The NCCAT collected three Krios datasets: ATAD2B-apo (9,541 micrographs), ATAD2B-ADP (12,945 micrographs), and ATAD2B-γATP (10,880 micrographs)(Figure 1, step 3). Since ATAD2B is an ATPase, we used γATP, which is a commonly used slowly hydrolyzable ATP analog to “trap” ATAD2B in a nucleotide-bound state (Cho et al., 2019, Cho et al., 2023, Wang et al., 2023). The best data was obtained for the γATP dataset, and we started processing (Figure 1, step 4). After pre-processing, 2D classification, and a few rounds of refinement to remove junk, an initial heptameric 3D map to 2.81 Å was obtained. As our very first attempt at solving a cryo-EM structure, generation of this 3D map was an exciting milestone for our lab. We were eager to start building the ATAD2B protein model into this novel heptameric map (Figure 1, step 5). The predicted AlphaFold structure for the ATAD2B monomer (AF-Q9ULI0-F1-v4) was manually docked into the initial map using ChimeraX. However, despite trying multiple manual docking strategies, such as docking each domain separately into the map, making dimers, trimers, tetramers of the ATAD2B model for docking, and running fitinmap commands in ChimeraX, we were unable to fit any region of ATAD2B into the density map.

With progress in data processing halted, we decided it was time to reach out to a cryo-EM expert for help. We shared our 2D class averages and initial 3D model while explaining the docking issues, thinking that maybe our collaborator would have a better idea for fitting the model into the map. The collaborator immediately identified our 2D class averages and 3D map as Gro-EL. Gro-EL is a ring-shaped *E. coli* chaperonin protein complex that helps mediate ATP-dependent polypeptide folding. It is an abundant, ubiquitous, and stress-induced protein that assists in folding proteins by interacting with incompletely folded polypeptides (Braig et al., 1994, Roh et al., 2017, Grimm et al., 1993, Fenton et al., 1994, Weissman et al., 1995). The presence of Gro-EL as a stress-induced protein made sense to us because we were pushing the upper limits of *E. coli* expression (Chae et al., 2017) with the 150 kDa ATAD2B protein complex, which has an oligomeric quaternary structure. However, we were puzzled by the mass spectrometry results, which returned every single band in addition to the liquid sample as ATAD2B. After reaching back out to the mass spectrometry core, we learned their analysis only searched our data against human protein databases. Once they opened up their search to include *E. coli* proteins, the analysis indicated the presence of Gro-EL in addition to ATAD2B.

Our analysis of the purified ATAD2B protein clearly indicate the sample is heterogeneous. A band is visible on the SDS-PAGE gel at the expected molecular weight for Gro-EL at 58 kDa (Figure 2b), which was confirmed via mass spectrometry. Gro-EL forms a stacked heptamer formed by 14 subunits, with a molecular weight of ~812 kDa. The hexameric ATAD2B is a AAA+ ATPase is expected to have a molecular weight of ~900 kDa. Therefore, these two proteins did not separate during analytical size exclusion chromatography, and Gro-EL co-purified with ATAD2B throughout the entire process. The mass spectrometry data indicated that ATAD2B and Gro-EL existed in similar quantities in the sample. We had the unfortunate realization that our protein purification, sample preparation, and data collection strategies (Figure 1, steps 1–3) were optimized for imaging Gro-EL particles instead of ATAD2B. However, we did have a minor amount of ATAD2B particles visible in the micrographs, so the next step was to return to data processing (Figure 1, step 4) to determine if we could salvage our cryo-EM data and identify more ATAD2B particles.

**Figure 3.**
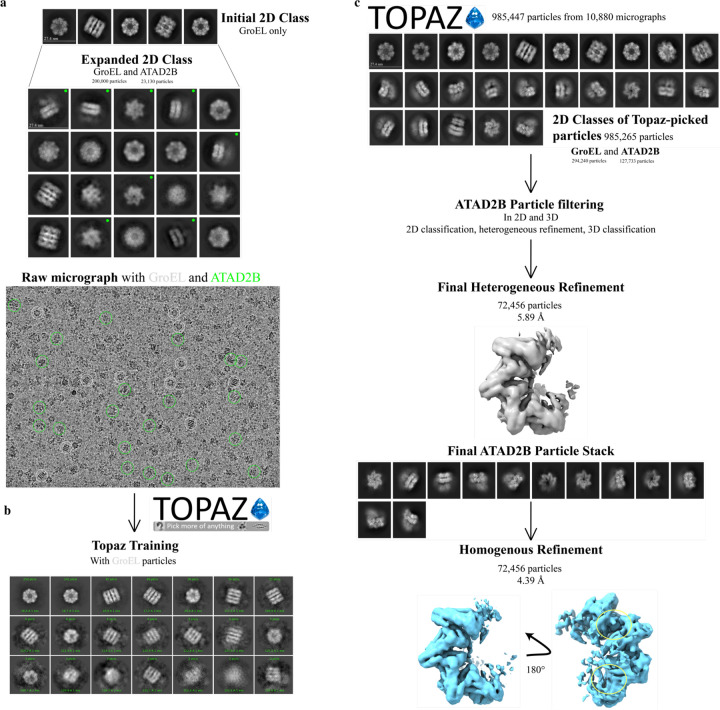
Cryo-EM data processing workflow to separate GroEL contaminants from ATAD2B particles. a) Initial 2D class averages from blob picker showed only heptameric GroEL particles (top). Upon further investigation, hexameric ATAD2B particles are evident, marked with a green circle (middle). Raw micrographs indicate the presence of both GroEL (white circle and unmarked particles) and ATAD2B particles (green circle). b) Topaz was trained to pick the GroEL particles in the raw micrographs. c) Topaz extract picked more ATAD2B particles than expected. 2D class averages of GroEL (top row) and ATAD2B particles (middle and bottom rows) are present in the same dataset and were identified in the 2D classes after Topaz extract. Heterogenous refinement was used to separate the ATAD2B and GroEL particles to prepare a final ATAD2B map.

Before completely starting over in the data processing workflow, we reanalyzed the initial extracted stack of particles (Figure 1, step 4). After a few expansions of 2D class averages, additional top and side hexameric 2D class averages of ATAD2B were found (Figure 3a). In total, this particle stack contained ~20,000 initial ATAD2B particles and ~200,000 initial Gro-EL particles. To find more ATAD2B particles, we went back a step further to explore different particle picking strategies. Altering the settings for the standard blob picker and template picker jobs yielded no improvements in ATAD2B particle number. However, we had the good fortune to attend a seminar on Topaz, which is a neural network based particle picking software that gets “trained” on a small subset of particles known to be your protein of interest, to then find these particles in the full dataset (Bepler et al., 2019). We started to use Topaz with our ATAD2B particle stacks, but because of how training works, it was nearly impossible to get an accurate picking model. To avoid introducing noise or false picks for ATAD2B, the higher quality and easier to find Gro-EL particles were used in the Topaz training step. We also decided it would be helpful to continue learning cryo-EM data processing with the Gro-EL particles, so we could apply this workflow later, once we had better quality data on ATAD2B. Incredibly, training Topaz on the Gro-EL particles not only picked Gro-EL particles, but also picked more ATAD2B particles than any other strategy tried thus far (Figure 3b). Topaz found 127,733 initial ATAD2B particles and 294,420 initial Gro-EL particles, indicating we had many ATAD2B particles in this sample despite the Gro-EL contamination. However, after filtering to remove junk particles from the Topaz picks, only <100,000 ATAD2B particles remained to use in reconstructions. Unfortunately, there were not enough ATAD2B particles present generate a map beyond mid-resolution (~4 Å) that would be needed to observe the details of nucleotide coordination in the ATAD2B binding pocket (Figure 3c).

We realized that it was time to return to the biochemistry (Figure 1, step 1) to obtain a more homogenous protein sample for cryo-EM structural investigation, without Gro-EL. There are a few protocols available to remove the Gro-EL contamination from a protein sample. One includes addition of unfolded bacterial lysate supplemented with ATP to try and outcompete Gro-EL from your protein of interest (Rohman & Harrison-Lavoie, 2000). We tried this purification method with a few rounds of optimization, however, Gro-EL still appeared at the same intensity on SDS-PAGE gels after purification. The final test was another cryo-EM screen and small overnight dataset collection, which confirmed that Gro-EL was present in the same amount prior to the addition of the unfolded bacterial lysate.

**Figure 4.**
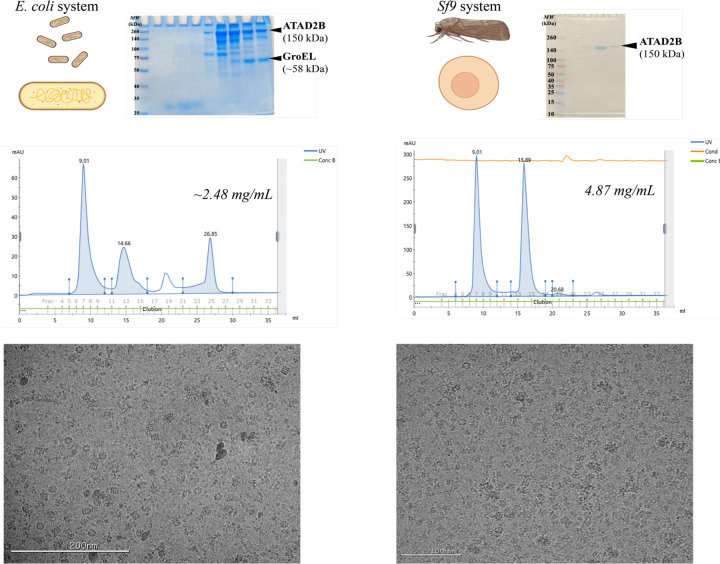
Sf9 Cell expression and purification procedure yields homogenous ATAD2B protein. Left top panel: Cartoon representation of *E. coli* expression and the corresponding 10% Coomassie-stained SDS-PAGE gel showing that the purified protein sample contains ATAD2B along with the GroEL contaminant. Left middle panel: Size-exclusion chromatography (Superdex 200 Increase 10/300 GL) profile of the purified sample displaying peaks corresponding to ATAD2B (at 9.01 mLs) and GroEL (at 14.66 mLs). Left bottom panel: Cryo-EM micrograph confirms the presence of heptameric GroEL contaminant in the purified sample. Right top panel: Cartoon representation of the *Sf9* expression system and the corresponding 10% Coomassie-stained SDS-PAGE gel showing that the purified protein sample contains more than 90% pure ATAD2B. Right middle panel: Size-exclusion chromatography (Superdex 200 Increase 10/300 GL) profile of the purified sample showing peaks corresponding to ATAD2B (at 9.01 mLs) and the cleaved GST tag (at 15.89 mLs). Right bottom panel: Cryo-EM micrograph confirms the presence of hexameric ATAD2B in the purified sample.

We had to decide if we should switch protein expression systems or collect enough cryo-EM data with the Gro-EL contaminant to obtain a higher resolution structure. In the γATP dataset, 10,880 micrographs were collected and less than 200,000 initial ATAD2B particles were observed before all of the junk was removed. Taking these numbers into consideration, we decided that it would require too much microscope time to make it worthwhile to continue with this sample. It was time to switch expression systems and return, again, to the biochemistry (Figure 1, step 1). In the literature, cryo-EM structures of ATAD2 homologs Abo1 and Yta7 were successfully determined from protein samples generated with the Sf9 insect cell expression system (Cho et al., 2019, Cho et al., 2023, Wang et al., 2023). We formed a new collaboration with Trybus Lab at the University of Vermont, who generously taught us how to express the ATAD2B protein using Sf9 inset cells. Within four months we obtained pure ATAD2B protein, free of Gro-EL, suitable for downstream cryo-EM studies (Figure 4). Obtaining a high-quality protein sample was essential for determining the structure of ATAD2B at near-atomic resolution, enabling us to characterize ligand coordination and protein–protein interactions that underpin its mechanism of action. This requirement parallels X-ray crystallography, where the quality of the crystal dictates the clarity of the diffraction data. Similarly, in cryo-EM, the purity and stability of the protein govern the resolution and reliability of the final structure–underscoring that sample preparation is the foundation for revealing atomic-level details in both techniques.

### Protein expression with chaperone and other common contaminants

The presence of chaperones and other contaminants are a frequent challenge in cryo-EM sample preparation that complicate the data collection and analysis pipeline (Table 4) (Zielinski et al., 2021). Molecular chaperones such as the chaperonin GroEL can co-purify with target proteins, as they bind unfolded or partially folded polypeptides, making them unwanted entities during purification (Weissman et al., 1995, Rutledge et al., 2022). Expression and purification of the AAA+ ATPase ATAD2B complex in *E. coli* resulted in GroEL contamination that co-purified with our target protein. Due to the similar sizes of both proteins, we did not realize the chaperone was present in our data until the 2D classification stage in data processing. Our attempts to separate the GroEL protein from ATAD2B during purification were unsuccessful. Therefore, we reverted to using insect cell lines of *Spodoptera frugiperda* (sf9) instead of bacterial protein expression systems for protein expression and purification. Moreover, based on our experiences it can be safely stated that effective cryo-EM studies require careful purification strategies, such as additional chromatography steps and nuclease treatments, as well as vigilant screening of grids to identify and minimize these potential contaminants.

To help future labs convert to this new technique, and avoid spending a lot of time troubleshooting removal of a contaminating protein, we would like to pass on what was helpful to us during this process. In Table 5, we have included a list of specialized data processing programs. The most common cryo-EM data processing software or platforms are found in Table 3, while a collection of helpful resources for learning various aspects of cryo-EM methods are mentioned in Table 1. We have included a list of common contaminants in Table 4 to help assess micrographs for their presence. We also encourage readers to investigate specific contaminants and why they may occur. Knowing when to switch expression systems will vary between labs. We chose to switch due to the amount of microscope time that would be required to collect a high-quality dataset. We needed to apply for time at the National CryoEM Center, which is a limited resource, and we felt that it would be better to create a better biochemical sample to collect a high quality dataset. Despite a higher upfront investment in time to switch expression systems, we decided moving to Sf9 cells would set the lab up future studies on large complexes that interact with chromatin. For more information on undesirable contaminations, and other issues that may impact image quality, such as ice thickness, contamination, preferred orientation, etc., we refer you to these reviews ((Tan et al., 2017),(Cianfrocco & Kellogg, 2020),(Neselu et al., 2023), and (Wang & Zimanyi, 2024)), and to Chapter 3 at cryoem101.org for guidance.

## 5. Data Processing Software for Single-Particle Cryo-EM Workflows

Processing of single-particle cryo-EM data is a computationally intensive process that relies on specialized software and advanced computing infrastructure. A good collaboration with the university IT department is essential. The data processing workflows, often utilizing software suites like CryoSPARC (Punjani et al., 2017), cisTEM (Grant et al., 2018, Lucas et al., 2021) and RELION (Scheres, 2012, Kimanius et al., 2016, Zivanov et al., 2022) show in (Table 3) generate massive amounts of data—from raw movies to intermediate files and final reconstructions—that demand high-performance computing (HPC) clusters with powerful GPUs, multi-terabyte storage, and high-speed networking. At the University of Vermont, support from the VACC staff was essential for building and maintaining this infrastructure, ensuring it can handle the unique demands of cryo-EM, such as parallel processing and large data transfers. Proper management of cryo-EM data is essential due to the large file sizes, and a clear strategy is essential in the storage of older datasets. As some downstream applications during data processing may need the raw data.

### Tutorial on data processing using the GroEL datasets to generate map reconstructions

**Figure 5.**
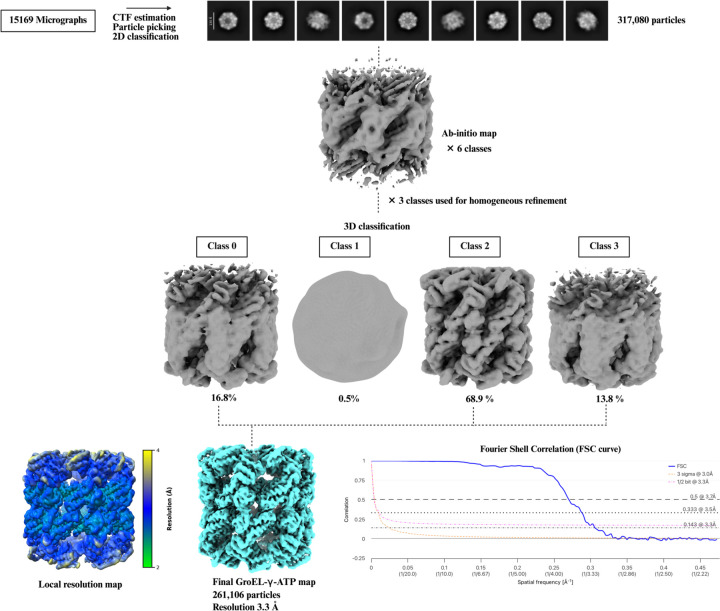
Single-particle cryo-EM data processing scheme for the GroEL-γATP bound complex. A total of 317,080 GroEL particles were selected following 2D classification and subjected to *ab initio* reconstruction into six classes. Three of these *ab initio* classes were retained for further processing and used to generate a homogeneous 3D map. Subsequent 3D classification yielded four classes, with one class identified as junk and excluded from further analysis. The remaining three classes were combined, resulting in a final reconstruction comprising 261,106 particles at a global resolution of 3.3 Å. Local resolution estimation was performed in cryoSPARC, and the resulting map was visualized using UCSF ChimeraX. The Fourier Shell Correlation (FSC) curve was generated using the EMDB validation tool (https://www.ebi.ac.uk/emdb/validation/fsc/).

Data processing for GroEL-γATP was performed in CryoSPARC v.4.5.31 (Punjani et al., 2017). All 15,169 movies were pre-processed through motion correction with patch motion correction. Patch contrast transfer function (CTF) estimation was done on the motion-corrected micrographs. This is essential for high-resolution reconstructions, as it enables more precise correction of image distortions. The two parameters of ice thickness and CTF fit were checked for the selection of 14,568 micrographs for further processing. The next step included picking particles from the micrographs. In CryoSPARC there are different methods to do this such as manual picking, blob picking, template and deep learning methods like Topaz (Bepler et al., 2019). The circular blob picker in CryoSPARC identifies particles in micrographs by detecting circular features of a specified size. It provides a fast, template-free method for initial particle selection. We used blob picker to pick 10,124,313 particles. The initially picked particles were filtered using defocus-adjusted power and pick scores, which help assess the quality and reliability of each pick. These metrics allow for the exclusion of low-quality or spurious particles before extraction. The selected particles were then extracted from the micrographs using a box size of 300 pixels, ensuring that each particle is fully captured for downstream processing. A stack of 3,056,751 particles was subjected to reference free 2D classification. This type of classification groups particles into a set number of classes by aligning and averaging them in-plane, improving the signal-to-noise ratio of each class average. This makes it easier to identify and remove poor-quality or contaminant classes. A total of 317,080 particles were used to generate six *ab-initio* classes. Three of the six *ab-initio* classes, comprising at total of 262,461 particles, resembled GroEL and were selected to generate a homogeneous reconstruction using D7 symmetry. A homogeneous map with D7 symmetry is a three-dimensional reconstruction in which all selected particles are refined together under the constraint of seven-fold dihedral symmetry, which can enhance resolution by leveraging the inherent symmetry of the complex. GroEL is known to exhibit D7 symmetry, as confirmed by previously published studies (Torino et al., 2023). To further analyze structural heterogeneity, the selected particles were divided into four distinct 3D classes without applying a focused mask, allowing for unbiased classification across the entire volume. This approach facilitates the separation of different conformational states and the removal of heterogeneous or junk particles. One of the resulting classes was identified as junk and excluded from further processing. The other three classes comprising 261,106 particles were combined to generate a final non-uniform map of 3.3 Å, according to the gold standard 0.143 FSC cutoff criteria. A scheme for the data processing is shown in Figure 5.

**Figure 6.**
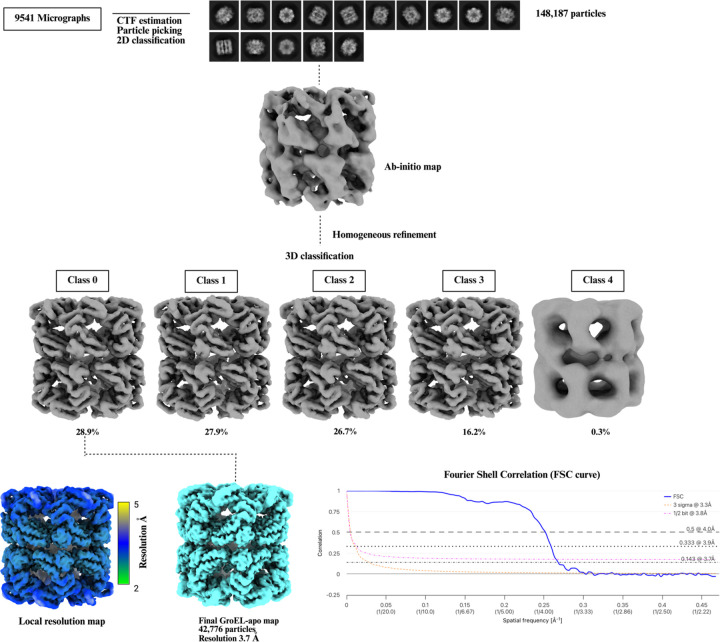
Single-particle cryo-EM data processing scheme for apo GroEL complex. A total of 148,187 GroEL particles were selected after 2D classification and subjected to *ab initio* reconstruction. Homogeneous refinement was performed on the resulting single class. Subsequent 3D classification yielded five classes; adopting a conservative approach, only the highest-quality class was selected for final map generation. The final reconstruction comprised 42,776 particles and had a final resolution of 3.7 Å. Local resolution estimation was performed in cryoSPARC, and the resulting map was visualized using UCSF ChimeraX. The Fourier Shell Correlation (FSC) curve was generated using the EMDB validation tool (https://www.ebi.ac.uk/emdb/validation/fsc/).

The GroEL-apo dataset comprising 9,541 micrographs was also processed using CryoSPARC v.4.5.31 (Punjani et al., 2017). Patch contrast transfer function (CTF) estimation was carried out on the motion-corrected micrographs. The processing scheme was similar to the GroEL-γATP dataset. However, in this dataset we first performed manual picking from selected micrographs to create a high-quality training set. This manually curated set of particles was then used to train a Topaz model (Bepler et al., 2019), enabling more accurate automated particle picking for the entire dataset. A total of 513,428 particles were extracted from the micrographs with a box size of 256 pixels. From the 2D classification, a total of 148,146 particles were used to generate one *ab-initio* class. This class was used to generate a homogeneous map with D7 symmetry and was further classified into 5 classes. The final map generated from non-uniform refinement had a resolution of 3.7 Å according to the 0.143 FSC cutoff criteria. The processing scheme is shown in Figure 6.

**Figure 7.**
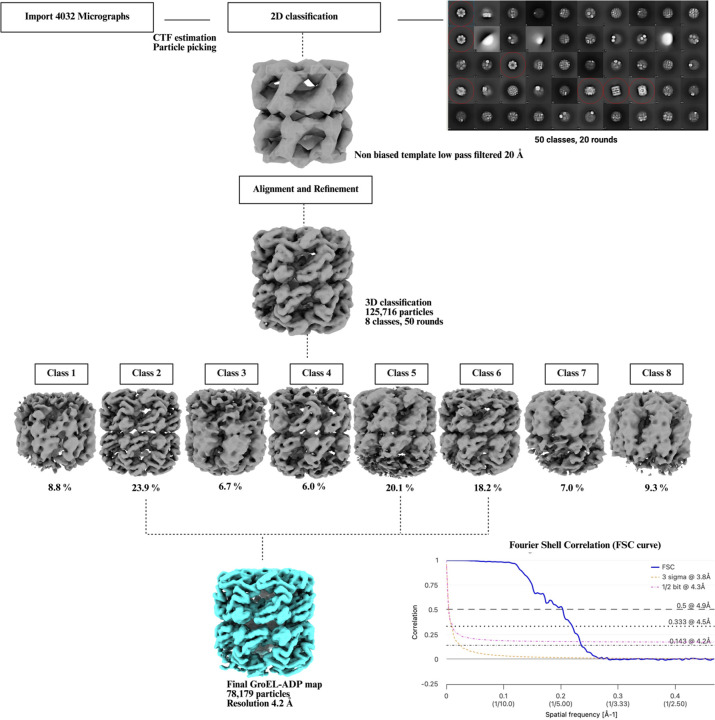
Single-particle cryo-EM data processing scheme for the GroEL-ADP bound complexes. The particle stack, comprising 125,716 GroEL particles selected from a 2D classification in cisTEM, was exported for further processing in FREALIGN. An initial alignment was performed using a low pass filtered GroEL template. Subsequently, the aligned particles underwent 3D classification over 50 rounds to sort them into distinct conformational states. However, no major confirmational changes were observed in the 3 best classes. These classes were combined to generate a final map of 4.2 Å. The two half-maps generated from the final 3D reconstruction were used to compute a Fourier Shell Correlation (FSC) curve by using the EMDB validation tool (https://www.ebi.ac.uk/emdb/validation/fsc/).

For the GroEL-ADP dataset we chose to use a different software called cisTEM (Grant et al., 2018) for data processing. 11,46,722 particles were picked using the *ab-initio* template picker a were subjected to reference free 2D classification. Particles in the 2D classes resembling GroEL were selected, and a stack of 158,542 particles was exported into FREALIGN (Grigorieff, 2016). The particle stack was binned 8× 4×, 2× using the command Ressample.exe. This binning of the particle stacks is done to reduce the computational processing time A non-biased low pass filtered (20 Å) map of GroEL was generated from the PDBID: 9C0C, and was used for initial particle alignment. The 2× binned stack was then 3D classified into 8 classes. The classes with the largest number of particles were combined to generate a final 1X map at a resolution of 4.2 Å. The processing scheme is shown in Figure 7.

## 6. Nuances of Model Building, Refinement, Cryo-EM Structure Validation and Deposition

Once the highest-quality reconstruction has been achieved, the next step is to interpret the cryo-EM density map by building an atomic model. This model may be derived from previously solved structures obtained through techniques such as X-ray crystallography or cryo-EM, or predicted using computational tools like AlphaFold. Regardless of the source, model building should be guided by prior biological knowledge and experimental context to ensure accurate interpretation. An integrated approach is essential, often combining structural, biochemical, and biophysical data to inform model construction and validation. Techniques such as mass spectrometry, cross-linking mass spectrometry (XL-MS), small-angle X-ray scattering (SAXS), nuclear magnetic resonance (NMR), size-exclusion chromatography (SEC), and SEC coupled with multi-angle light scattering (SEC-MALS) can provide valuable insights into the composition, organization, and conformational states of the molecular complex.

In X-ray crystallography diffraction patterns obtained from highly ordered crystals yield cryo-EM maps with sharp, well-defined features. This clarity enables confidence in the positions of individual atoms. However, a major obstacle in this process is the “phase problem.” While the intensities of diffracted X-rays can be measured directly, the phase information which is important for constructing the cryo-EM map is not captured in the experiment. Consequently, specialized experimental techniques or computational approaches must be employed to estimate or recover these missing phases to solve the crystal structures (Adams et al., 2009).

In contrast, cryo-EM reconstructs three-dimensional electrostatic potential maps from thousands of two-dimensional images of individual, flash-frozen molecules captured in various orientations. These maps often exhibit lower signal-to-noise ratios and variable resolution across different regions of the molecule, particularly in flexible regions (Punjani & Fleet, 2023). However, unlike X-ray crystallography, cryo-EM captures phase information directly from the experimental data rather than relying on model-derived phases. This can lead to more accurate and detailed representations in high-resolution regions and reduces model bias during interpretation. Consequently, model building in cryo-EM frequently involves fitting known protein fragments or predicted structures into the density, with continuous tracing of the polypeptide chain being more challenging, especially at lower resolutions, and often relying on iterative refinement to improve the fit and to identify secondary structure elements (Venien-Bryan et al., 2017).

### Model building of GroEL in different nucleotide bound states

The initial steps of model building for X-ray crystallography and cryo-EM differ significantly due to the nature of the data (Terwilliger et al., 2020). However, after the initial fitting of the starting model in the final map (Figure 1, step 5), these two techniques become similar. For our three GroEL structures, we used PDBIDs 8BL7 and 9C0C as starting models. Here, we will focus on the model building of GroEL-γATP, scheme shown in Figure 5. The initial model was rigid body fitted in the final map using UCSF ChimeraX (Pettersen et al., 2004). As the name indicates, it is just placing the initial rigid model in the map based on optimal position and coordinates without changes to the model’s shape. The coordinates for the γATP ligand (AGS) were taken from PDBID: 5DAC and was rigid body fitted into the map. Further manual adjustments were done using Coot (Crystallographic Object-Oriented Toolkit) (Emsley & Cowtan, 2004), which is a molecular graphics program for building and validating macromolecular models. Our GroEL model was then structurally refined in the cryo-EM map using phenix.real_space_refine in Phenix (Liebschner et al., 2019). This program refines the atomic coordinates while simultaneously enforcing good stereochemistry, such as ideal bond lengths, angles, and proper protein backbone and side-chain conformations.

**Figure 8.**
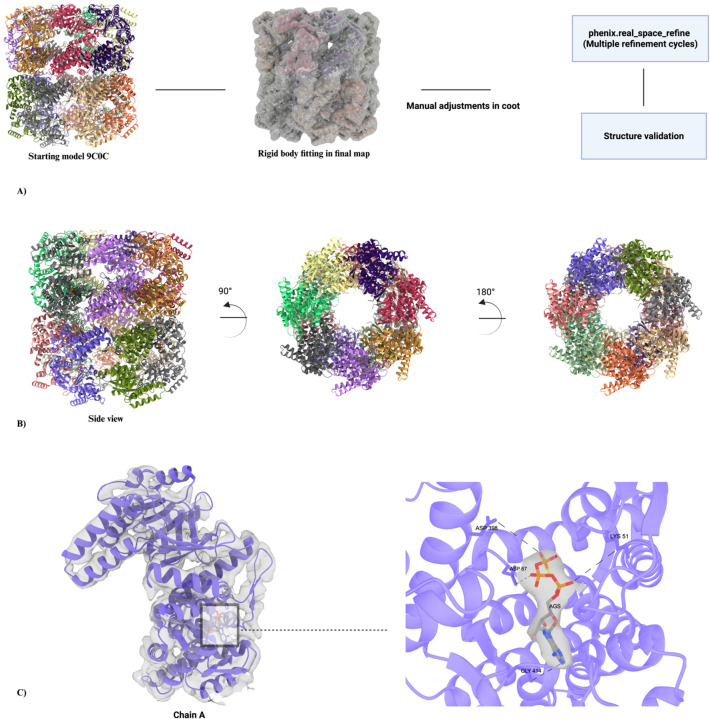
Model building and structural details of the GroEL-γATP bound complex. a) Schematic workflow of the model building and refinement process. The starting model for structural refinement was PDBID: 9C0C (GroEL-apo). Initially, the model was fitted in the final map using UCSF ChimeraX, followed by manual adjustments in COOT. Iterative refinement cycles were performed in Phenix (phenix. real_space_refine), and structure validation was carried out using MolProbity. The resulting models have favorable stereochemical parameters, including minimal deviation from ideal bond lengths and angles, and a low number of macromolecular backbone outliers. b) A side view of the multimeric GroEL complex is shown on the left, with each of the 14 subunits colored differently. The middle and right panels show top and bottom views of the complex respectively, to highlight the overall architecture and subunit organization. c) The left panel displays subunit A of GroEL bound to γATP (purple), with a boxed region indicating the ATPase active site. The right panel highlights this region in detail, showing the molecular surface enveloping the bound γATP. Key residues involved in ATP hydrolysis are labeled including: LYS 51 (involved in ATP phosphate binding), ASP 87 and ASP 97 (coordinate the essential Mg^2+^ ion and help position water for nucleophilic attack), ASP 398 (contributes to allosteric signaling and conformational changes), and GLY 414 (provides structural flexibility for proper positioning of catalytic residues).

During the refinement steps, secondary restraints files were generated for the model and were used in subsequent refinement cycles. The restraints prevent the model from deviating from physically and chemically plausible conformations. Without these restraints, the refinement software would be at risk of overfitting the model to noise or artifacts in the cryo-EM map, leading to a geometrically unreliable structure. During the refinement, correlation coefficients (model-to-map fit in Phenix) were closely monitored to prevent overfitting of the models in their respective maps. The high correlation coefficients for all three structures confirmed an excellent fit between the final atomic models and the experimental density maps. Separately, validation of the resulting models indicated favorable stereochemical parameters, including minimal deviation from ideal bond lengths and angles, suggesting high quality throughout the model geometry. Structural details of GroEL-γATP are also shown in Figure 8, with closer views of the nucleotide binding site of chain A. Figures were generated using UCSF ChimeraX (Pettersen et al., 2004) and PyMOL (version 2.3.1, Schrödinger, LLC) (DeLano, 2002).

### Structure Validation

During model validation Phenix does give a wide range of statistics for validation, as MolProbity is incorporated into the software (Davis et al., 2007). However, the model can also be validated in the MolProbity web browser at https://molprobity.biochem.duke.edu/. An overview of data collection and validation statistics of all three structures are shown in Table 6. Here we will go through the validation procedure used for the GroEL-γATP complex. During model refinement in Phenix (Adams et al., 2010), global refinement statistics were monitored after each cycle to assess model quality and convergence. Upon completion of the refinement process, the final model-to-map cross-correlation coefficient (CCC) was 0.83, indicating a strong agreement between the atomic model and the experimental cryo-EM map. For geometry restraints, root mean square deviation (RMSD) for bonds and angles, quantifies how much the model’s geometry deviates from established ideal values. The low RMSD values for bonds (0.003 Å) and angles (0.599°) demonstrated that the model’s geometry was close to the expected ideal values. In addition, Z-scores (0.223 and 0.390, respectively) further confirm this, as they are very low and well within an acceptable range.

The all-atom clashscore for the GroEL-γATP was 3.1, which indicated very few steric clashes between atoms. The Ramachandran plot is a graphical way to visualize the energetically favored and allowed backbone conformations of amino acid residues in a protein. It plots the two main dihedral angles of the protein backbone, phi (ϕ) and psi (ψ). The Ramachandran plot had 0.00% outliers and a very high percentage of residues in the “favored” region (97.4% in this case). The rotamer outliers are amino acid side-chain conformations that are found in energetically unfavorable positions. Their presence could indicate potential errors in the model, as side chains typically adopt a limited set of stable conformations known as rotamers to minimize steric clashes. There were 0.1% rotamer outliers, which indicated favorable side chain conformations.

After reviewing the validation statistics, we checked each residue of our model to ensure its quality and adherence to stereochemical principles. This process helps identify potential errors or issues that could lead to future validation problems. Afterwards, we started the PDB validation, for this purpose the refined model (GroEL-γATP) and final map were uploaded to https://validate-rcsb-2.wwpdb.org/. The PDB validation report provides an overall summary, using color-coded flags to highlight potential issues. A green flag indicates a metric is within expected ranges; yellow suggests a potential issue, and red flags a significant problem. Another metric in the validation report was the Q-score (Pintilie et al., 2020), which quantifies the resolvability of individual atoms in a 3D density map. It measures how well the map values around a specific atom’s position correlate with an ideal, well-resolved Gaussian-like function, with a score of 1 indicating a perfect fit and a value closer to 0 or negative indicating poor resolvability or a poor fit, for all 14 chains in the our GroEL-γATP model, the Q-score was 0.443.

### Structure deposition and repositories for cryo-EM data

Best practices for cryo-EM data deposition emphasize transparency, reproducibility, and community access. Final atomic models should be deposited in the Protein Data Bank (PDB), while corresponding 3D density maps must be submitted to the Electron Microscopy Data Bank (EMDB). For raw image data, deposition in the Electron Microscopy Public Image Archive (EMPIAR) is strongly encouraged, as it enables method development, validation, and training of new algorithms. These repositories provide persistent identifiers and standardized validation reports, ensuring compliance with funding, journal, and community guidelines. Using the OneDep system, models and maps can be deposited to the PDB and EMDB simultaneously through a single deposition workflow, streamlining the process and ensuring consistency across related datasets. Detailed instructions for deposition, including file formats and validation requirements, are available on the respective websites:

- **PDB**: https://www.wwpdb.org/
- **EMDB**: https://www.ebi.ac.uk/emdb/
- **EMPIAR**: https://www.ebi.ac.uk/empiar/

Following these practices aligns with the NIH 2023 Data Management & Sharing Policy and the NSF Public Access and Data Management & Sharing Plan requirements, promoting timely public access to publications and supporting data, and strengthening the transparency and reproducibility of federally funded research. It also supports the broader structural biology community in benchmarking and advancing the field.

## 7. Conclusions and Future Directions

Our transition from X-ray crystallography to single-particle cryo-EM provided critical insights into the structural characterization of ATAD2B, a large AAA+ ATPase and bromodomain-containing protein involved in chromatin regulation. This journey highlighted both the transformative potential of cryo-EM and the practical challenges associated with adopting this technique, including sample heterogeneity, co-purification of chaperones such as GroEL, and the steep learning curve for data processing and computational infrastructure. Despite these obstacles, we successfully established a workflow that integrates biochemical optimization, advanced vitrification strategies, and state-of-the-art image processing tools.

Looking forward, cryo-EM is poised to redefine structural biology by enabling visualization of macromolecular assemblies in unprecedented detail and in increasingly native contexts. Advances in detector technology, sample preparation, and computational methods—including machine learning-driven particle picking, heterogeneity analysis, and integrative modeling—will accelerate data processing and interpretation. Furthermore, the expansion of cryo-electron tomography and correlative workflows will bridge the gap between isolated complexes and cellular environments, offering a holistic view of molecular machines in action.

Together, these developments position cryo-EM to deliver not only higher resolution but higher information content: dynamic ensembles, contextualized structures, and integrative models aligned with cellular function. For the crystallographic community, the opportunity is clear—leveraging cryo-EM alongside crystallography and complementary biophysical approaches will deepen mechanistic understanding across scales, inform structure-guided discovery, and establish robust, community-vetted standards for the next decade of structural biology.

## Figures and Tables

**Table 1:** Helpful Resources for Getting Started in Cryo-EM

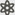 Name	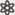 Brief Summary	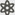 Quick Link	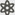 Expertise Needed
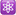 ** Cryo-EM (NIH Common Fund)**	The home page for the National Cryo-EM Centers in California, New York, and Oregon	**Broadening Access to high-resolution cryo-electron microscopy and tomography**	Resource
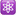 **Cryo-EM Centers List (PNCC)**	The Pacific Northwest CryoEM Center has compiled a global list of cryo-EM instrumentation and resources.	**Cryo-EM Service Centers, A Working List**	Resource
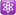 **Cryo-EM Centers World Wide Map**	The Pacific Northwest CryoEM Center has compiled a global list of cryo-EM instrumentation and resources.	**Cryo-EM in a Worldwide Map**	Resource
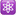 ** National Center Resources** YouTube Link: NCCAT Short Courses	Fantastic series of lectures on theory with sections directly involved in data processing from experts in the field. Includes links to lectures and handouts from each year a short course is held.	**NCCAT Classes, Workshops and short courses Homepage**	Beginner Intermediate Advanced
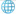 **cryoEDU**	Information on the theory and emphasis on why to process a certain way. Provides examples of what to look for.	**Hands-on cryo-EM educational tool**	Beginner Intermediate
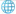 cryoEM101.org	Explains concepts in an accessible, hands-on way with example images and situations.	**Cryo-EM 101 website**	Beginner
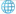 **CryoSPARC**	A tutorial/how-to guide to use one of the data processing software programs.	**Getting Started with CryoSPARC Introductory** **Tutorial**	Beginner Intermediate Advanced
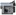 ** Grant Jensen YouTube Videos:** Direct YouTube Link: Grant Jensen Cryo-EM Videos	Long-form lectures on foundational theory with PDF handouts of the slides. Very technical, but very rewarding.	**Caltech Getting Started in Cryo-EM**	Beginner Intermediate Advanced
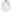 ** Merit Badge Homepage**	Informative videos to watch for when preparing to begin your hands-on experience.	**US Cryo-EM Center Merit Badges**	Intermediate
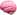 ** ThermoFisher Cryo-EM Learning Center**	Multi-resource area for the latest news, articles, information, etc.	**Life Sciences Electron Microscopy Resource Center**	Beginner
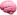 ** ThermoFisher Cryo-EM University**	Great resource for highly informative videos regarding each step of the cryo-EM workflow.	**Cryo-EM University**	Beginner
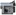 ** Yale University Fred Sigworth’s Cryo-EM Principles** Direct YouTube Link: Cryo-EM Principles by Fred Sigworth	Long-form lectures on foundational theory with PDF handouts. More videos expected…	**Cryo-EM Principles Home Page**	Beginner Intermediate Advanced

**Table 2.** Factors to Consider During Cryo-EM Sample Preparation

Category	Condition / Instrument
**Sample Type**	Choose proteins or complexes suitable for cryo-EM (e.g., over 50 kDa, non-crystallizable)
**Initial Screening**	Perform negative staining to assess particle integrity, homogeneity, and aggregation.
**Buffer Optimization**	Optimize salt (typically 150 −500 mM NaCl), glycerol (typically 0–5%), and pH (typically 6.5–8.5); consider adding: – Reducing agents (DTT, TCEP)– Detergents (e.g., OG) to reduce aggregation or preferred orientation– Cofactors or ligands (e.g., ATP, Mg^2+^) to stabilize functional complexes.
**Protein Concentration**	Test multiple concentrations to avoid aggregation (too high) or sparsity (too low).
**Vitrification Devices**	Compare methods (Vitrobot, Leica GP2, Chameleon) to minimize air–water interface damage.
**Grid Type Selection**	Use different grids (Quantifoil, UltrAuFoil, Graphene-coated) to optimize particle orientation and distribution.
**Ice Quality Control**	Maintain a clean, dry environment; use dried tools, LN₂ transfer boxes, and wear face masks to prevent frost and contamination.

**Table 3.** Core computation requirements for popular cryo-EM image processing platforms

Computational Requirements			
Component (Minimum Requirements)	CryoSPARC(Punjani et al., 2017)	RELION(Scheres, 2012)	cisTEM(Grant et al., 2018)
**CPU**	≥16 cores (Intel i9 / AMD Ryzen)	≥16 cores (Intel i9 / AMD Ryzen)	≥16 cores (Intel i9 / AMD Ryzen)
**RAM**	64 GB	64 GB	64 GB
**GPU**	1× NVIDIA RTX 3060 / A2000	1× NVIDIA RTX 3060 / A2000	Not Supported
**Fast Storage (SSD)**	1–2 TB NVMe SSD	1–2 TB NVMe SSD	1–2 TB NVMe SSD
**Bulk Storage**	≥10 TB HDD or NAS	≥10 TB HDD or NAS	≥10 TB HDD or NAS
**Network**	1 Gbps	1 Gbps	1Gbps
**Operating System**	Linux (Ubuntu, CentOS, RHEL)	Linux (Ubuntu, CentOS, RHEL)	Linux (Ubuntu, CentOS, RHEL)
	cryo-EM Platforms		
**Platform**	**Type**	**Notes**	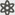 **Quick Link**
**CryoSPARC Cloud**	Cloud	Fully cloud-hosted by Structura Biotechnology Inc.	
**Open Science Grid (OSG)**	Shared Facility HPC Cluster	National academic HPC resource	https://osg-htc.org
**COSMIC**^**2**^ **Platform**	Shared Facility HPC Cluster	Centralized national resource	https://www.cosmic2.org
**AWS Batch/EC2**	Cloud	Commercial cloud infrastructure	https://aws.amazon.com/products/
**ScipionCloud**(Cuenca-Alba et al., 2017)	Hybrid (Cloud + Shared/Local HPC)	Web interface; jobs can run on cloud or connect to local/university cluster	

**Table 4.** Common Cryo-EM Contaminants

Protein contaminant	Notes
**ArnA, SlyD and AcrB** (Andersen et al., 2013, Caliseki et al., 2025)	Frequently contaminate samples with His-tagged fusion proteins expressed in *E. coli* due to their natural histidine-rich sequences.
**GroEL / GroES** (Nain et al., 2022)	Very common chaperonin contaminant from *E. coli* expression systems.
**Ferritin** (Wenborn et al., 2015)	Typically encountered in samples derived from animal tissues or produced using mammalian expression systems.
**Ribosomal proteins / fragments** (Bolanos-Garcia & Davies, 2006, Singh et al., 2020, Amunts et al., 2014)	Can be found in protein samples purified from bacterial or mammalian cells.
**Heat shock proteins (Hsp70, Hsp90, sHsps)**(Carr et al., 2025, Bolanos-Garcia & Davies, 2006)	These chaperones often bind unfolded proteins and co-purify with them.
**Affinity tag fragments (Glutathione S-transferase (GST) / Maltose-binding protein (MBP)** (Waugh, 2011)	Tag remnants are common contaminants in proteins purified using affinity tags.

**Table 5.** Specialized Modular Programs for Data Processing

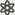 Name	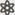 Brief Description	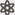 Quick Link
AlphaFold (Jumper et al., 2021)	AI system for predicting protein 3D structures from amino acid sequences.	https://alphafold.ebi.ac.uk/
ChimeraX (Pettersen et al., 2021)	Molecular visualization program for displaying and analyzing 3D biological structures, including atomic models and cryo-EM maps	https://www.cgl.ucsf.edu/chimerax/
cisTEM (Grant et al., 2018)	Software for single-particle cryo-EM image processing and reconstruction.	https://cistem.org/
CryoDRGN (Zhong et al., 2021)	Deep learning for heterogeneous cryo-EM data.	https://cryodrgn.csail.mit.edu/
cryoSPARC (Punjani et al., 2017)	Software platform for processing and analysis of cryo-EM data	https://cryosparc.com/
crYOLO (Wagner et al., 2019)	Neural network-based particle picker for cryo-EM micrographs.	https://cryolo.readthedocs.io/en/stable/
DynaMight (Schwab et al., 2024)	A tool to model continuous conformational changes in cryo-EM datasets	https://github.com/3dem/DynaMight/
EMAN2 (Tang et al., 2007)	Suite for single-particle reconstruction and electron microscopy image processing.	https://blake.bcm.edu/emanwiki/EMAN2
Phenix (Adams et al., 2010, Afonine et al., 2018, Liebschner et al., 2019)	Software suite for macromolecular structure determination by X-ray crystallography and cryo-EM.	https://phenix-online.org/
ModelAngelo (Jamali et al., 2024)	Automated atomic model building for cryo-EM maps.	https://github.com/3dem/model-angelo/
Topaz (Bepler et al., 2019)	Machine learning particle picking for cryo-EM.	https://cb.csail.mit.edu/topaz/

**Table 6.** Details of structural refinement of all three GroEL structures/models.

Data collection and processing	GroEL-ATP PDBID: 9YKC EMD-73044	GroEL-ADP PMID: 9YNJ EMD-73200	GroEL-apo PDBID: 9YKE EMD-73045
**Magnification**	81000	81000	81000
**Voltage (kV)**	300	300	300
**Electron exposure (e–/Å2)**	64	64	52.5
**Defocus range (μm)**	−0.8 µm to −2.5 µm	−0.8 µm to −2.5 µm	−0.8 µm to −2.5 µm
**Pixel size**	1.058	1.069	1.069
**Symmetry imposed**	D7	D7	D7
**Final map particles**	261,106	78,179	42,776
**Final map resolution**	3.3 Å	4.2 Å	3.7 Å
**FSC threshold**	0.143	0.143	0.143
**Refinement details**			
**Model used (PDB code)**	8BL7	9C0C	9C0C
**Resolution of starting model**	4.40 Å	3.41 Å	3.41 Å
**Correlation Coefficient (cc_mask)**	0.83	0.87	0.87
**Map sharpening B factor (Å^2^)**	−60	−100	−40
**MolProbity score**	1.30	1.38	1.29
**Clash score**	3.1	4.0	3.02.9
**Poor rotamers**	0.1 %	0 %	0 %
**Ramachandran plot**			
**Favored (%)**	97.4%	98.0%	97.2%
**Allowed (%)**	2.6%	2.0	2.8%
**Disallowed (%)**	0%	0%	0%
**CaBLAM outliers**	2.3%	1.4%	1.6%
**Q-score**	0.443	0.305	0.396
